# Use of Short Tandem Repeat Sequences to Study *Mycobacterium leprae* in Leprosy Patients in Malawi and India

**DOI:** 10.1371/journal.pntd.0000214

**Published:** 2008-04-09

**Authors:** Saroj K. Young, Jorg M. Ponnighaus, Suman Jain, Sebastian Lucas, Sujai Suneetha, Diana N. J. Lockwood, Douglas B. Young, Paul E. M. Fine

**Affiliations:** 1 Clinical Research Unit, Department of Infectious and Tropical Diseases, London School of Hygiene and Tropical Medicine, London, United Kingdom; 2 Karonga Prevention Study, Chilumba, Karonga District, Malawi; 3 Blue Peter Research Centre, LEPRA Society, Hyderabad, India; 4 Department of Histopathology, Kings College London School of Medicine, St Thomas' Hospital, London, United Kingdom; 5 Centre for Molecular Microbiology and Infection, Division of Investigative Sciences, South Kensington Campus, Imperial College London, London, United Kingdom; 6 Infectious Disease Epidemiology Unit, Department of Epidemiology and Population Sciences, London School of Hygiene and Tropical Medicine, London, United Kingdom; Institut Pasteur, France

## Abstract

**Background:**

Inadequate understanding of the transmission of *Mycobacterium leprae* makes it difficult to predict the impact of leprosy control interventions. Genotypic tests that allow tracking of individual bacterial strains would strengthen epidemiological studies and contribute to our understanding of the disease.

**Methodology/Principal Findings:**

Genotyping assays based on variation in the copy number of short tandem repeat sequences were applied to biopsies collected in population-based epidemiological studies of leprosy in northern Malawi, and from members of multi-case households in Hyderabad, India. In the Malawi series, considerable genotypic variability was observed between patients, and also within patients, when isolates were collected at different times or from different tissues. Less within-patient variability was observed when isolates were collected from similar tissues at the same time. Less genotypic variability was noted amongst the closely related Indian patients than in the Malawi series.

**Conclusions/Significance:**

Lineages of *M. leprae* undergo changes in their pattern of short tandem repeat sequences over time. Genetic divergence is particularly likely between bacilli inhabiting different (e.g., skin and nerve) tissues. Such variability makes short tandem repeat sequences unsuitable as a general tool for population-based strain typing of *M. leprae*, or for distinguishing relapse from reinfection. Careful use of these markers may provide insights into the development of disease within individuals and for tracking of short transmission chains.

## Author Summary

Molecular typing has provided an important tool for studies of many pathogens. Such methods could be particularly useful in studies of leprosy, given the many outstanding questions about the pathogenesis and epidemiology of this disease. The approach is particularly difficult with leprosy, however, because of the genetic homogeneity of *M. leprae* and our inability to culture it. This paper describes molecular epidemiological studies carried out on leprosy patients in Malawi and in India, using short tandem repeat sequences (STRS) as markers of *M. leprae* strains. It reveals evidence for continuous changes in these markers within individual patients over time, and for selection of different STRS-defined strains between different tissues (skin and nerve) in the same patient. Comparisons between patients collected under different circumstances reveal the uses and limitations of the approach—STRS analysis may in some circumstances provide a means to trace short transmission chains, but it does not provide a robust tool for distinguishing between relapse and reinfection. This encourages further work to identify genetic markers with different stability characteristics for incorporation into epidemiological studies of leprosy.

## Introduction

Implementation of standardised multidrug regimens in the 1980s and 1990s has had a major impact on global leprosy prevalence, through shortening the duration of treatment. While it was reasoned that effective treatment of individual patients would reduce the spread of *Mycobacterium leprae* within communities, there is little evidence that the elimination programme has had a significant impact on disease incidence [1]. Continued controversies over global trends in the epidemiology of leprosy highlight gaps in our knowledge of the basic mechanisms of infection transmission and pathogenesis of this poorly understood disease [2]. While direct spread by aerosol or contact with infected individuals is thought to be the major route for dissemination of *M. leprae*, a role for zoonotic or environmental reservoirs cannot be excluded.

Publication of the genome sequence of an *M. leprae* isolate in 2001 revealed an organism which has undergone an extensive evolutionary process of gene decay [3]. Subsequent genetic studies have revealed that current human isolates of *M. leprae* from around the world show very little variation [4]. Identification of a limited number of informative single nucleotide polymorphisms (SNPs) allowed the elucidation of a high-level global phylogeny for *M. leprae*, but the extraordinary degree of sequence conservation has so far precluded application of SNP-based typing in the analysis of local epidemiology and mapping of transmission chains.

In contrast to the extreme conservation uncovered by SNP analysis, several researchers have reported a highly dynamic pattern of variation in copy number of short tandem repeat sequences in the *M. leprae* genome [5]–[8]. Using these loci, considerable variability was observed amongst panels of isolates within restricted geographical areas [5], [8]–[11]. In most, though not all, cases, the pattern of polymorphisms was conserved during passage in experimentally infected nude mice [12]. Inability to grow *M. leprae* in axenic culture has prevented quantitative measurement of the frequency in which changes in copy number are generated.

The aim of the present study was to assess whether differences in copy number of short tandem repeats provides information that is informative about the transmission of *M. leprae*. To evaluate this, we have applied molecular typing to leprosy patient biopsies collected in the course of epidemiological studies carried out in northern Malawi and in Hyderabad, India. This has allowed two different evaluations: firstly to assess the stability of the genotype within individuals; and secondly to assess transmission within leprosy multi-case households.

## Methods

### Patients

The Malawi (Karonga) series included 43 biopsies which had been collected for diagnostic purposes from 17 leprosy patients at the same or different times. The patients were identified from records of the Karonga Prevention Study [13]–[15]. The skin biopsies, taken by 6 mm punch, and partial thickness nerve biopsies, taken from enlarged sensory nerves, had been fixed in formol-Zenker and sent to the UK for histopathological analysis. Biopsies were read independently, and assigned classifications (TT = polar tuberculoid; BT = borderline tuberculoid; BB = Borderline; BL = borderline lepromatous; LL = Polar lepromatous; IND = Indeterminate) [16] by S Lucas. All patients gave informed consent for biopsy collection, and ethical permissions for this study were obtained from the Malawi Health Sciences Research Committee and from the Ethics Committee of the London School of Hygiene and Tropical Medicine.

The Indian (Hyderabad) series included 20 biopsies from 20 patients from eight families with more than one patient. All these patients were diagnosed at the outpatients clinic of the Blue Peter Research centre (BPRC), Hyderabad, India. All biopsies had been taken by 6 mm punch for diagnostic purposes, fixed in 10% buffered formal saline, embedded in paraffin and processed for histopathology [5]. Ethical approval was obtained from the local BPRC Ethics Committee and informed consent was obtained from all the subjects involved in the study.

### DNA extraction from paraffin-embedded tissues

Ten sections of 5 µm thickness were collected in a separate vial for each sample. A separate blade was used to cut each block in order to avoid cross contamination. The sections were deparaffinised prior to extraction. 100 µl of extraction buffer (90 µl of 0.5 M EDTA pH 8.0, 0.5% SDS+10 µl Proteinase K–QIAGEN) was added to each sample, mixed and incubated with constant agitation at 56°C overnight. Each sample was centrifuged at 223×g for 5 min and 100 µl of the supernatant then transferred into a tube containing 0.5 ml PB buffer (QIAGEN PCR purification kit) and thereafter DNA extraction was done according to the manufacturer's instructions [17].

### Genotyping

A total of seven repeat loci were examined. These were identified by analysis of the *M. leprae* genome sequence (of an isolate derived from South India) [18] and from previous publications [5]–[7]. Table 1 provides a list of PCR primers used. All PCR products were analysed by gel purification and sequencing to determine repeat copy numbers as described previously. DNA purified from armadillo-grown *M. leprae* supplied by Dr P Brennan through the NIH Leprosy Contract (http://www.cvmbs.colostate.edu/mip/leprosy/index.html) was used as a positive control.

**Table 1:** Short tandem repeat loci and PCR primers employed in this study, and their size in base pairs (bp).

Locus	Genome location	Repeat size	PCR primers	Amplicon size	Repeat sequence	Translated sequence
ML2172	2583816–2583839	3 bp	F: ATCAACGCTGCGGTTTCGCAG	151 bp	AGT	(intergenic)
			R: ATATGCATGCCGGTGGTGTGCT			
ML2334	2785432–2785494	3 bp	F: CGTTGGGTTCGATCGAATCGA	131 bp	TTC	(intergenic)
			R: GCACGCCGACGGGAATAAGT			
ML1505	1816857–1816892	6 bp	F: ATGGCAACCATCTGGACCTACT	173 bp	GCACCT	Pro-Ala repeat
			R: GGCCGCCGAACTGATCAA			
ML1182	1381661–1381723	12 bp	F: GTCAATGAGGTCGGGTTAGC	157 bp	GAGGTTGTTGAG	Glu-Val-Val-Glu repeat
			R: GCGGTACGACAGTAGCTTCC			
ML0058	73077–73139	21 bp	F: ATTACCAATTTGCAAGGAGTGCTCAGCT	186 bp	CTTGATCAAGTTGATTCCTGG	(pseudogene)
			R: GAAATTTGGCGGCCATATGCC			
ML2469	2945487–2945555	23 bp	F: GTCGCCCGGATACTGTTAAA	188 bp	ATAATACTGTAGTGAACGACATC	(intergenic)
			R: TAAATCCGCTCCCAAATCTT			
ML2418	2893248–2893761	25 bp	F: GTCGAGACGATCCTTGTTGC	189 bp	AGAATTTACCGGCGTTCA*ATAAGAA	(pseudogene)
			R: TGCGCTCCTAGTTGAGCTTC			

*: single nucleotide mismatch in second copy

PCR was performed on a Hybaid Express thermal cycler in a final volume of 25 µl using the ‘Hot-Start’ Excite Core Kit (BioGene) according to the manufacturer's instructions. After an initial denaturation step (10 min at 95°C), 45 cycles of amplification were performed as follows: denaturation at 95°C for 15s, annealing at 58°C for 40s, and extension at 72°C for 30s. A final extension was performed at 72°C for 2 min. PCR products were initially screened by electrophoresis in 2% (w/v) agarose gels. For sequencing, products were separated on 2% (w/v) low melting point agarose (Invitrogen) and bands were excised with a sterile scalpel blade and purified using a GeneClean DNA isolation kit (Q-BIO gene). Cycle sequencing was performed on a PE 2700 system with ABI Big Dye 3.1 Terminator Ready Reaction Kit (Applied Biosystems) according to the manufacturer's protocol, with subsequent analysis on an ABI 3730 Genetic Analyzer.

## Results

### Polymorphic repeat loci

Based on inspection of the genome sequence of *M. leprae*, we selected a panel of seven loci containing short tandem repeat elements ranging from 3 to 25 base pairs. To assess the extent of polymorphism of these loci among the Malawi patients, we amplified and sequenced the corresponding products from all 43 skin and nerve biopsies taken from 17 individual patients (Table 2). We were able to determine repeat copy numbers for almost all of the loci, even from biopsies which had been scored as polar tuberculoid and with no bacilli seen by microscopy. A total of 26 different copy number combinations were observed from the 43 samples.

**Table 2:** Distribution of copy numbers of seven different short tandem repeat loci in the 43 Malawi biopsies.

Number of Copies	Short tandem repeat locus						
	ML2172	ML2334	ML1505	ML1182	ML0058	ML2469	ML2418
**1**					8		
**2**					29	43	43
**3**					6		
**4**				4			
**5**				39			
**6**			32				
**7**	1		9				
**8**	18						
**9**	19	4					
**10**	4	8					
**11**	1						
**12**		11					
**13**		1					
**14**		2					
**15**		4					
**16**		11					
**17**		2					
***M. leprae*** ** (reference)**	8 copies	10 copies	7 copies	5 copies	2 copies	2 copies	2 copies
***M. leprae*** ** (sequence)**	9 copies	21 copies	7 copies	5 copies	3 copies	3 copies	5 copies

Numbers in the table give the number of biopsies with each particular number of copies of each of seven tandem repeat loci. The “reference” strain was derived from a patient in USA; the “sequence” strain was derived from a South Indian patient (Cole et al, 2001).

Polymorphism was more extensive in the case of the shorter repeat elements. The two longer repeats, ML2469/70 (23 bp) and ML2418 (25 bp), were uniformly present in two copies in all of the Malawi samples. This differs from the copy numbers in the sequenced isolate (3 and 5 copies respectively) but is identical to a recent sample of armadillo derived *M. leprae* DNA provided by the NIH Reference Facility at Colorado State University. For subsequent analysis of the Malawi samples, we used only the five shorter repeat loci.

### Consistency of genotype within individual patients

To assess the utility of genotypic analysis for mapping of transmission, we first assessed whether repeat copy number could vary between samples from a single individual, by analysis of Malawi biopsies taken at a single timepoint from different anatomical sites or tissues, and biopsies taken from the same individual at different times.

#### Multiple skin biopsies

For seven patients, we tested paired skin biopsies taken at the same time from different skin lesions (Table 3). For six of the cases, the genotypes of the paired biopsies were identical across all five loci. For the seventh patient (a 37 year old with borderline lepromatous leprosy), the genotype differed at four of the five loci (the biopsies were taken from lesions on the right and left scapular regions of the patient's back). This suggests that, either the genotype of the infecting isolate can undergo multiple changes during the evolution of the infection within an individual, or that the patient had been co-infected by multiple “strains” of differing genotype.

**Table 3:** Comparison of copy numbers of short tandem repeat loci in multiple skin biopsies taken at the same time, from different lesions, from individual patients in Malawi.

Patient number	Age (yrs)	Site/classification	Short tandem repeat loci				
			ML2172	ML2334	ML1505	ML1182	ML0058
2	60	Skin/BT	9	10	6	4	3
		Skin/BT	9	10	6	4	3
3	44	Skin/BT	8	12	6	5	2
		Skin/BT	8	12	6	5	2
4	55	Skin/LL	9	16	6	5	1
		Skin/LL	9	16	6	5	1
7	46	Skin/TT	9	9	6	5	2
		Skin/TT	9	9	6	5	2
10	38	Skin/BL	8	14	7	5	2
		Skin/BL	8	14	7	5	2
16	35	Skin/BL	9	9	6	5	2
		Skin/BL	9	9	6	*nd*	2
17	37	Skin/BL	9	10	6	4	1
		Skin/BL	11	17	6	5	3

Histopathological classifications in column 3.

#### Comparison of skin and nerve biopsies

Biopsies taken from skin and nerve lesions at a single timepoint were analysed from seven patients (Table 4). In contrast to the paired skin biopsies, differences in copy number were seen between all of the nerve-skin pairs. For three patients the copy number changed by one in a single locus, one patient had a change in two loci, and for the remaining three patients skin and nerve samples differed at three of the five loci.

**Table 4:** Comparison of copy numbers of short tandem repeat loci from multiple biopsies taken at a single timepoint from different anatomical locations, from individual patients in Malawi.

Patient number	Age (yrs)	Site/classification	Short tandem repeat loci				
			ML2172	ML2334	ML1505	ML1182	ML0058
1	52	Skin/BT	9	10	7	5	2
		Nerve/BT	9	16	6	5	2
5	63	Skin/BT	8	12	7	5	3
		Nerve/BL	8	12	6	5	3
6	35	Skin/ BT	9	15	6	5	2
		Nerve/BL	10	15	6	5	2
9	31	Skin/IND	8	16	6	5	2
		Nerve/BL	8	16	6	5	1
11	37	Skin/BT	9	16	6	4	2
		Nerve/BT	10	16	6	5	2
		Nerve/BT	10	16	6	5	2
12	23	Skin/BT	9	12	6	5	1
		Nerve/BT	7	10	6	5	2
13	52	Skin/BT	8	13	7	5	1
		Nerve/BL	8	10	6	5	2

Histopathological classifications in column 3.

#### Comparison over time

Biopsies taken at multiple timepoints were analysed for four Malawian patients for whom clinical analysis had been repeated as a result of relapse or reactivation episodes (Table 5). Changes in copy number were observed in successive skin biopsies from all these patients. For patient 8, serial skin biopsies taken four times over a period of five years each differed at one or more loci.

**Table 5:** Comparison of copy numbers of short tandem repeat loci from multiple biopsies taken from the same anatomical location at different times, from individual patients in Malawi.

Patient number	Date (Age in yrs)	Site/classification	Short tandem repeat loci				
			ML2172	ML2334	ML1505	ML1182	ML0058
4	05/90 (55)	Skin/LL	9	16	6	5	1
	11/94 (59)	Skin/BT	9	12	7	5	2
5	01/87 (63)	Nerve/BL	8	12	6	5	3
	01/88 (64)	Nerve/BT	8	12	6	5	2
8	07/89 (53)	Skin/BL	8	15	6	5	2
	10/89 (53)	Skin/BL	10	15	6	5	2
	10/90 (54)	Skin/**?** *	8	12	6	5	2
	07/94 (58)	Skin/BT	9	12	6	5	2
15	07/90 (45)	Skin/IND	9	16	6	5	2
	08/93 (48)	Skin/IND	8	12	6	5	3

The initial biopsies of patients 4 and 5 are included in Tables 3 and 4, respectively.

*: The biopsy from patient 8 in 1990 was considered by the histopathologist to show “normal epidermis”.

### Genotype analysis in multicase families

Copy numbers at three of the repeat loci were analysed for a panel of skin biopsies taken from the Indian patients from eight multicase families (Table 6). With three exceptions (see C, D and F), members of these families lived together in the same household residence. Identical genotypes were found in all the cases in six of the families. Two individuals from family “G” lived in same residence but had bacilli which differed at a single locus. In family “D”, three individuals shared an identical genotype, one differed at a single locus, and one differed at all three loci. Interestingly, the two individuals with bacilli of different genotypes were the oldest in the entire series, and the one which differed at all three loci was the most distantly related (mother of a daughter in law) and lived in a different residence.

**Table 6:** Comparison of copy numbers of short tandem repeat loci in biopsies from patients in multicase families in India.

Household	Relationship between patients	Age	Class	Year	Short tandem repeat loci		
					ML2172	ML2334	ML0058
**A**	Father	38	LL	2001	8	14	nd
	daughter	14	BL	2001	8	14	nd
**B**	Brother	10	BT	2000	8	12	2
	sister	12	BT(*rrxn*)	2000	8	12	2
**C**	Mother	28	LL(*rel*)	2004	8	17	2
	Brother *	17	LL	2000	8	17	2
	son	14	BL	2003	8	17	2
**D**	Mother	61	BL(*rrxn*)	2003	9	16	2
	son	25	LL(*enl*)	2004	9	13	2
	daughter-in-law	20	BT(*rrxn*)	2004	9	13	2
	grandson	6	BL(*rrxn*)	2004	9	13	2
	mother of d-i-law *	48	BL	2004	8	14	1
**E**	Husband	25	BT(*rrxn*)	2003	8	13	2
	Wife	22	BT	2004	8	13	2
**F**	brother-in-law	31	LL(*enl*)	2000	8	13	2
	brother-in-law **	26	LL(*enl*)	2001	8	13	2
**G**	Husband	25	LL(*enl*)	2003	10	10	2
	Wife	20	BT(*rrxn*)	2004	12	10	2
**H**	Brother	21	LL(*enl*)	2002	8	16	3
	Brother	20	BT	2004	8	16	3

All members of each family group lived in the same household residence except for individuals marked ^*^, who lived in separate residences nearby, and individual marked ^**^ who lived in another household, in another village. Abbreviations: *rrxn = *reversal reaction (type 1 reaction); *rel = *relapse; *enl = *erythema nodosum leprosum (type 2 reaction).

## Discussion

Our findings in the Malawi patients are consistent with previous publications demonstrating extensive polymorphism in the copy number of short tandem repeat sequences of *M. leprae* [5]–[8]. Comparison with the relative paucity of SNP polymorphisms suggests that *M. leprae* may have acquired a specific lesion in the mechanisms required for maintenance of fidelity during replication of repeat sequences, possibly as one of the consequences of the overall pattern of gene decay in this organism [18]. Comparison of different repeat loci indicates that changes are most extensive in the case of very short repeat motifs comprising only 2 or 3 base pairs. This is consistent with the reported absence of polymorphism in longer repeat sequences resembling the mycobacterial interspersed repeat units (MIRU) that have proved useful in typing of *M. tuberculosis* [18].

While we cannot exclude the possibility that changes in repeat copy number have potentially selectable phenotypic consequences, inspection of the predicted changes does not indicate any obvious biological significance. Three of the repeats are located outside of coding regions. The 6bp ML1505 locus introduces a variable number of Pro-Ala repeats within a conserved hypothetical protein; the 12bp ML1182 locus encodes a Glu-Val-Val-Glu repeat in a member of the PPE protein family; the ML0058 21bp repeat, ML2469 23bp repeat and the ML2418 25bp repeat are located in pseudogenes.

Analysis of multiple Malawi biopsies collected at the same time reproduced our previous finding among Indian patients [5], with significantly greater genotypic differences between *M. leprae* samples collected from skin and nerve than between multiple skin samples from the same patient (7/7 skin-nerve versus 1/7 skin-skin patients; *p<0.01*). This could indicate that the patients with more than one *M. leprae* genotype had been infected by more than one isolate, and that the isolates were tissue specific, or that progression of the infection results in expansion of different bacterial populations in different anatomical sites. To explore further the possibility that the genotype may change during disease progression, we analysed serial samples from four patients from Malawi. In no patient were the serial isolates identical. While again we cannot totally exclude the possibility of multiple infections, the variation in copy number observed over time suggests that the dominant genotype can undergo changes within a single individual.

The potential occurrence of genotypic variation within individual patients points to a need for considerable caution in any application of this type of analysis to tracking of transmission between individuals. On the other hand, when we analysed repeat copy numbers at three loci for individuals with a high probability of sharing a transmission link as a result of living in the same household, in Hyderabad, India, we observed a strong concordance in bacterial genotype. This is consistent with our earlier observation in this same population [5].

The homogeneity in genotypes between individuals within households in the Hyderabad series contrasts with the differences observed between individuals in Malawi, and also with the differences observed within individuals over time and between tissues in the Malawi series. We offer three comments on these patterns. First, as only three loci were examined in the Hyderabad series, versus five in the Malawi patients, our ability to detect differences was lower for the Indian than for the Malawian series. This may have increased the apparent homogeneity of the Hyderabad household sets. Second, the Indian series included more individuals towards the lepromatous pole than did the Malawi series. Perhaps the relatively unrestrained growth of *M. leprae* in lepromatous patients allows selection of dominant clones, and/or these patients were infected with large numbers of genetically identical bacilli within their household environments. Third, the Malawi patients were appreciably older than the Indian patients. Though tuberculoid disease is thought to have a shorter incubation period than lepromatous disease, this age difference, and the fact that leprosy has declined rapidly in Malawi in recent years [13],[14], means that the Malawian patients are likely to have been infected for longer than the Indian patients, which could explain the genetic divergence of the *M. leprae* populations between individuals. The fact that the two oldest patients in the Hyderabad series had isolates which differed from those of their household contacts is consistent with this.

Taken together, our findings suggest that genotyping of *M. leprae* on the basis of short tandem repeat copy numbers may provide insights into disease progression within individual patients and, when used with care, may assist in analysis of short and recent transmission chains. Current evidence indicates that it does not provide a robust assay to distinguish recent transmission from relapse, or reinfection from reactivation, in the way that molecular tools have proved useful for study of the epidemiology of tuberculosis. It remains possible that further repeat loci will be identified as having an intermediate stability suitable for wider transmission tracking.
